# Heterozygous *PGM3* Variants Are Associated With Idiopathic Focal Epilepsy With Incomplete Penetrance

**DOI:** 10.3389/fgene.2020.559080

**Published:** 2020-10-15

**Authors:** Xiao-Rong Liu, Wen-Jun Bian, Jie Wang, Ting-Ting Ye, Bing-Mei Li, De-Tian Liu, Bin Tang, Wei-Wen Deng, Yi-Wu Shi, Tao Su, Yong-Hong Yi, Wei-Ping Liao

**Affiliations:** Key Laboratory of Neurogenetics and Channelopathies of Guangdong Institute, Department of Neurology of the Second Affiliated Hospital of Guangzhou Medical University, of Neuroscience, Province and the Ministry of Education of China, Guangzhou, China

**Keywords:** whole-exome sequencing, congenital disorder of glycosylation, immunodeficiency, *PGM3* gene, idiopathic focal epilepsy

## Abstract

**Introduction:**

Idiopathic focal epilepsy (IFE) is a group of self-limited epilepsies. The etiology for the majority of the patients with IFE remains elusive. We thus screened disease-causing variants in the patients with IFE.

**Methods:**

Whole-exome sequencing was performed in a cohort of 323 patients with IFE. Protein modeling was performed to predict the effects of missense variants. The genotype–phenotype correlation of the newly defined causative gene was analyzed.

**Results:**

Four novel heterozygous variants in *PGM3*, including two *de novo* variants, were identified in four unrelated individuals with IFE. The variants included one truncating variant (c.1432C > T/p.Q478X) and three missense variants (c.478C > T/p.P160S, c.1239C > G/p.N413K, and c.1659T > A/p.N553K), which had no allele frequency in the gnomAD database. The missense variants were predicted to be damaging and affect hydrogen bonds with surrounding amino acids. Mutations Q478X, P160S, and N413K were associated with benign childhood epilepsy with centrotemporal electroencephalograph (EEG) spikes. P160S and N413K were located in the inner side of the enzyme active center. Mutation N553K was associated with benign occipital epilepsy with incomplete penetrance, located in the C-terminal of Domain 4. Further analysis demonstrated that previously reported biallelic *PGM3* mutations were associated with severe immunodeficiency and/or congenital disorder of glycosylation, commonly accompanied by neurodevelopmental abnormalities, while monoallelic mutations were associated with milder symptoms like IFE.

**Conclusion:**

The genetic and molecular evidence from the present study implies that the *PGM3* variants identified in IFE patients lead to defects of the *PGM3* gene, suggesting that the *PGM3* gene is potentially associated with epilepsy. The genotype–phenotype relationship of *PGM3* mutations suggested a quantitative correlation between genetic impairment and phenotypic severity, which helps explain the mild symptoms and incomplete penetrance in individuals with IFE.

## Introduction

Idiopathic focal epilepsy (IFE), also named as localization-related idiopathic epilepsy (G40.0 in ICD-10 2016, WHO), is a common group of self-limited childhood-onset epilepsies. It contains two major subtypes, i.e., benign childhood epilepsy with centrotemporal electroencephalograph (EEG) spikes (BECTS, OMIM# 117100) and benign occipital epilepsy (BOE, OMIM# 132090). BECTS is affecting 0.2% of the population (Strug et al., 2009), while the prevalence of BOE is unknown but is considered to affect around 6% of children aged 1–15 years (Weir et al., 2018). Although both BECTS and BOE are generally regarded as autosomal dominant inheritance with age-dependent penetrance, rare causative genes are identified in the patients. Recent studies have shown that several genes, including *GRIN2A*, *ELP4*, *SRPX2*, and *DEPDC5*, are associated with BECTS (Strug et al., 2009; Lemke et al., 2013; Lal et al., 2014; Reinthaler et al., 2014) *GRIN2A* mutations were regarded as the most relevant causative gene for BECTS but accounted for only 4.9% of the patients with BECTS (Lemke et al., 2013). So far, no causative gene has been reported to be associated with BOE. Thus, the etiology for majority of patients with IFE remains unknown.

In this study, we performed whole-exome sequencing (WES) in a cohort of patients with IFE. Four novel heterozygous variants in *PGM3* were identified. Previously, *PGM3* mutations were associated with immunodeficiency-23 (IMD23, OMIM# 615816) and/or congenital disorder of glycosylation (CDG) that is occasionally accompanied by seizures. Our further analysis showed that previously reported homozygous and compound heterozygous mutations in *PGM3* were associated with severe immunodeficiency and glycosylation disturbance, while monoallelic mutations were potentially associated with mild phenotypes like IFE, suggesting that *PGM3* is potentially a candidate causative gene of epilepsy.

## Materials and Methods

### Subjects

A total of 323 subjects with IFE were recruited in the Epilepsy Center of the Second Affiliated Hospital of Guangzhou Medical University in China from January 2014 to 2019. These subjects were epilepsy patients with partial seizures and/or focal discharges but without acquired causes. Their EEGs showed focal abnormalities with features of idiopathic epilepsies, including shifting, bilateral, or multiple focal discharges with normal backgrounds. Eligible subjects had a clinical diagnosis of IFE according to the International League Against Epilepsy (ILAE-1989, 2006, and 2017) (Commission on Classification and Terminology of the International League Against Epilepsy, 1989; Engel, 2006; Scheffer et al., 2017) after appropriate investigations, including long-term video electroencephalography (VEEG), brain MRI, cognitive and behavioral evaluation, and neurometabolic testing. All of the subjects were followed up for at least 1 year.

The studies adhered to the guidelines of the International Committee of Medical Journal Editors with regard to patient consent for research or participation and received approval from local ethics committees of the participating hospitals. The Ethics Committee of the Second Affiliated Hospital of Guangzhou Medical University provided ethics approval.

### Whole-Exome Sequencing

Genomic DNA was extracted from peripheral blood using a QuickGene DNA whole blood kit (Fujifilm, Tokyo, Japan). Blood samples of parents and siblings were used for linkage and segregation analysis. Trio-based whole-exome sequencing was conducted on the Illumina HiSeq 2500/4000 platform by BGI-Shenzhen (Shenzhen, China). Paired-end reads with a length of 90bp were generated by massive parallel sequencing with more than 125 times average depth and more than 98% coverage of the target region. The raw data were aligned to the reference human genome (GRCh37) using the Burrows–Wheeler Alignment (BWA). Potential pathogenic variants were derived by stepwise filtering. Population-based filtration removed common variants presenting a minor allele frequency ≥0.005 in the gnomAD database, except for those previously reported to be associated with disease in the Human Gene Mutation Database (HGMD) and/or the Online Mendelian Inheritance in Man database. Potential pathogenic variants were flagged if predicted as damaging by multiple *in silico* programs (Supplementary Table 1). Inheritance-based filtration filtered variants based on family history and possible inheritance models. Assessment of pathogenicity of the variants was performed following the ACMG guidelines (Richards et al., 2015). Conservation of mutated positions was evaluated using sequence alignment of different species. All *PGM3* variants were annotated based on the transcript ENST00000512866.1. Sanger sequencing was used to validate the positive findings and the variant origination.

### Mutation Analysis

To evaluate the damaging effect of candidate variants, protein modeling was performed using the Iterative Threading ASSEmbly Refinement (I-TASSER) software (Zhang, 2009; Yang and Zhang, 2015). The confidence of each modeling was quantitatively measured by a C-score of 0.14. The three-dimensional structures were shown using PyMOL 1.7.

In an attempt to evaluate the genotype–phenotype correlation, *PGM3* mutations and their related phenotypes were systematically retrieved on the PubMed database till November 2019.

## Results

### Identification of *PGM3* Variants

**FIGURE 1:**
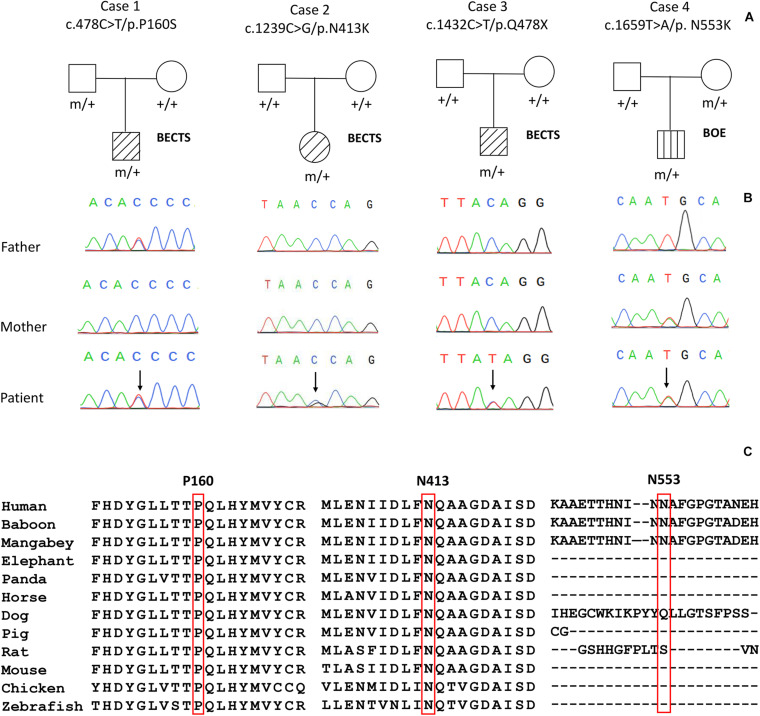
Genetic data on the patients with idiopathic focal epilepsy with *PGM3* mutations. **(A)** Pedigrees of the four cases with *PGM3* mutations and their corresponding phenotypes. **(B)** DNA sequence chromatogram of the *PGM3* mutations. Arrows indicate the positions of the mutations. **(C)** The amino acid sequence alignment of the three missense mutations shows that residues P160 and N413 are highly conserved across vertebrates. Residue N553 is less conserved and does not appear in the transcript of several species.

Four variants in the *PGM3* gene (OMIM^∗^ 172100) were identified, including one nonsense variant (c.1432C > T/p.Q478X) and three missense variants (c.478C > T/p.P160S, c.1239C > G/p.N413K, and c.1659T > A/p.N553K). Three variants (P160S, N413K, and Q478X) were identified in the cases with BECTS, and one variant (N553K) was in the case with BOE. Variants N413K and Q478X were *de novo*, while variants P160S and N553K originated from their asymptomatic parents, respectively (Figures 1A,B). These variants in *PGM3* had no allele frequency in the gnomAD database. Variant P160S was predicted to be damaging or probably damaging by all used *in silico* programs, variant N413K was predicted to be damaging or probably damaging by 11 out of 16 prediction tools, and variant N553K was predicted to be damaging by SIFT and fitCons (Supplementary Table 1). The amino acid sequence alignment showed that residues P160 and N413 were highly conserved across vertebrates. Residue N553 was less conserved in vertebrates and did not appear in the transcript of several species (Figure 1C). Variant Q478X was estimated as a pathogenic variant, and variant N413K was estimated as a likely pathogenic variant by ACMG assessment, while variants P160S and N553K were uncertain significant variants. All cases had no other pathogenic or likely pathogenic variants in genes known to be associated with seizure disorders (Wang et al., 2017).

### Structural Alteration of *PGM3* Protein

**FIGURE 2:**
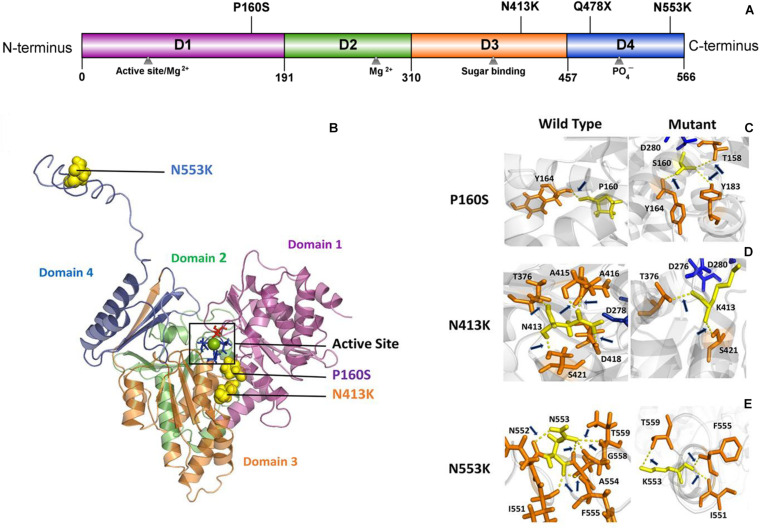
Schematic illustration of mutation and interactions with surrounding amino acids. **(A)** Linear schematic of the PGM3 structure and the location of identified *PGM3* mutations. PGM3 is composed of Domains 1–4. Domain 1 is colored purple, Domain 2 in green, Domain 3 in yellow, and Domain 4 in blue. Mutation P160S locates in Domain 1, mutation N413K locates in Domain 3, and mutations Q478X and N553K in Domain 4. **(B)** Schematic illustration of the location of mutations in the three-dimensional structure of PGM3. The active site is depicted in black frame. The residue S64 (colored red) locates in Domain 1. Residues D276, D278, and D280 (colored blue) locate in Domain 3. The green sphere represents the magnesium (Mg) ion. The yellow spheres represent the mutations. Mutations P160S and N413K locate within the active center. Mutation N553K is a distance away from the active center. **(C–E)** Hydrogen bond changes of mutants P160, N413, and N553. Arrows index the hydrogen bonds.

PGM3 contains four domains (Domains 1–4) (Figure 2A). The catalytic cleft is formed by the active serine loop in Domain 1, the metal-binding loop in Domain 2, the sugar-binding loop in Domain 3, and the phosphate-binding loop in Domain 4 (Nishitani, 2006). The truncating variant Q478X is located in Domain 4 and would lead to haploinsufficiency. Variant P160S is located in Domain 1, and variant N413K is located in Domain 3. The three-dimensional structural model of *PGM3* indicated that residues P160 and N413 lay in the inner side of the enzyme active center and were expected to be more “deleterious.” Variant N553K, located in Domain 4, which lay in the C-terminal of Domain 4 and a distance away from the enzyme active center (Figure 2B), potentially led to mild damage effect. The molecular effects of the missense variants analyzed by protein modeling using I-TASSER showed that all the three missense variants led to the alterations of hydrogen bonds and may affect the protein steric configuration. Originally, residue P160 formed a hydrogen bond with Y164. When proline was replaced by serine at residue 160, additional two hydrogen bonds were formed with Y183 and T158 (Figure 2C). Residual N413 formed five hydrogen bonds with T376, A415, A416, D418, and S421, respectively. When asparagine was replaced by lysine, the hydrogen bonds with A415, A416, and D418 were destroyed, and only the hydrogen bonds with T376 and S421 were kept (Figure 2D). Residue N553 originally formed hydrogen bonds with I551, N552, A554, G558, and T559, respectively. When the asparagine was replaced by lysine at 553, the hydrogen bonds with N552, A554, and G558 were destroyed, and a new hydrogen bond with F555 was formed instead (Figure 2E).

### Clinical Features of the Cases With *PGM3* Variants

**FIGURE 3:**
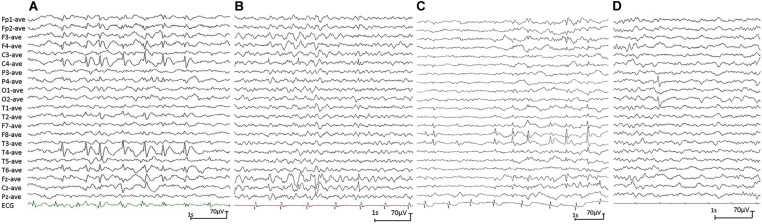
Electroencephalograph (EEG) changes during non-rapid-eye-movement sleep in the cases with idiopathic focal epilepsy with *PGM3* mutations (referential montage with average reference was used in all EEGs). **(A)** Interictal EEG of case 1 showed right centrotemporal spike and slow waves (obtained at the age of 8 years). **(B)** Interictal EEG of case 2 showed right central spike and slow waves (at the age of 3 years). **(C)** Interictal EEG of case 3 showed bilateral temporal spike and slow waves (at the age of 12 years). **(D)** Interictal EEG of case 4 showed right occipital spike waves (at the age of 3 years).

All affected cases showed childhood-onset focal epilepsy. Table 1 summarized the main clinical features of the four cases with *PGM3* variants. The cases with variants P160S, N413K, and Q478X were diagnosed as BECTS. The three cases started seizures at preschool or school age with generalized tonic–clonic seizure (GTCS) during sleep. The GTCSs occurred with approximately one to two attacks per year. No developmental delay, intellectual or speech disability, or visual abnormalities were observed. Their electroencephalograph (EEG) showed unilateral or bilateral independent centrotemporal discharges, dominantly during sleep (Figures 3A–C). The brain MRIs were normal. They all got seizure-free by low-dose monotherapy of valproic acid or lamotrigine. The case with variant N553K was diagnosed as BOE. He was a 3-year-old boy with normal development and had two GTCSs at the age of 2 years. The EEG obtained at the age of 3 years showed right occipital discharges (Figure 3D). His brain MRI was normal. He was seizure-free for 9 months without any treatment of antiepileptic drugs. Except seizures, no manifestation of immunodeficiency and CDG was observed in the four cases.

**TABLE 1:** Clinical and genetic features of the cases with *PGM3* mutations.

	Case 1	Case 2	Case 3	Case 4
*PGM3* mutation	c.478C > T/p.P160S	c.1239C > G/p.N413K	c.1432C > T/p.Q478X	c.1659T > A/p.N553K
Origin	Paternal	*De novo*	*De novo*	Maternal
Epilepsy syndrome	BECTS	BECTS	BECTS	BOE
Gender	Male	Female	Male	Male
Present age	12 yr	4 yr	17 yr	3 yr 9 mo
Age of seizure onset	7 yr 10 mo	3 yr	12 yr 4mo	1 yr 6 mo
Seizure types	sGTCS	GTCS	GTCS	GTCS
Frequency of seizure	1/yr	1/yr	2/mo	2/yr
Family history of seizure	None	None	None	None
Intelligence	Normal	Normal	Normal	Normal
Developmental delay	No	No	No	No
Speech	Normal	Stutter	Normal	Normal
Behavior	Normal	Salivation	Normal	Normal
Vision	Normal	Normal	Normal	Normal
Movement disorder	None	None	None	None
EEG discharges	Rt centrotemporal	Rt centroparietal	Bilateral temporal	Rt occipital
Brain MRI	Normal	Normal	Normal	Normal
Treatment	VPA 10 mg/kg/day	LTG 5 mg/kg/day	VPA 250 mg/bid	NA
Seizure outcome	Free for 3 yr	Free for 0.5 yr	Free for 4 yr	Free for 1.5 yr
MAF in gnomAD	None	None	None	None
ACMG assessment	Uncertain significant (PM2 + PP3)	Likely pathogenic (PS2 + PM2 + PP3)	Pathogenic (PVS1 + PS2 + PM2)	Uncertain significant (PM2)

**GTCS, generalized tonic–clonic seizures; sGTCS, secondary GTCS; BECTS, benign epilepsy of childhood with centro-temporal spikes; BOE, benign occipital epilepsy; NA, not available; yr, year; mo, month; VPA, valproic acid; LTG, lamotrigine; MAF, minor allelic frequency; Rt, right.**

### Genotype–Phenotype Correlation of *PGM3* Mutations

Previously, 18 mutations in *PGM3* were identified (Table 2). Sixteen out of 18 mutations, including 10 homozygous mutations and 3 compound heterozygous mutations, were identified in 16 families, involved 28 individuals (Sassi et al., 2014; Stray-Pedersen et al., 2014; Zhang et al., 2014; Lundin et al., 2015; Bernth-Jensen et al., 2016; Pacheco-Cuellar et al., 2017; Lundin et al., 2018; Abolhassani et al., 2019). All the individuals carrying biallelic mutations had the symptoms of immunodeficiency and/or congenital disorder of glycosylation (CDG). The neurological features included cognitive disability (12/28), developmental delay (12/28), seizures (10/28), characteristic facies (8/28), and hypotonia (4/28). The mutations included 11 missense, two small deletions, one small insertion, one splicing mutation, and one gross deletion including the whole gene of *PGM3.*

**TABLE 2:** Summary of neurological features in the cases/families with *PGM3* mutations.

Allele 1	Allele 2	Phenotype	Age of onset	Cognitive disability	Developmental delay	Seizure	Characteristic facies	Hypotonia	References
**Homozygous mutations**									
c.248T > C/p.L83S	c.248T > C/p.L83S	IMD	3–4 mo	1/4	3/4	–	4/4	1/4	Sassi et al., 2014
c.631G > C/p.D211H	c.631G > C/p.D211H	IMD	1–8 yr	NA	NA	NA	NA	NA	Abolhassani et al., 2019
c.737A > G/p.N246S	c.737A > G/p.N246S	CDG, IMD	Birth	2/2	2/2	–	2/2	–	Stray-Pedersen et al., 2014; Bernth-Jensen et al., 2016
c.877 + 3A > G	c.877 + 3A > G	IMD	3 w	–	–	–	–	–	Lundin et al., 2018
c.891T > G/p.D297E	c.891T > G/p.D297E	IMD, NI	10–12 yr	2/3	3/3	2/3	–	1/3	Zhang et al., 2014
c.965T > C/p.I322T	c.965T > C/p.I322T	IMD	13 mo	–	–	–	–	–	Lundin et al., 2015
c.1019_1021delAAG/p.E340del	c.1019_1021delAAG/p.E340del	IMD	1–7 mo	2/4	2/4	1/4 (3 yr)	–	2/4	Sassi et al., 2014
c.1135T > C/p.F379L	c.1135T > C/p.F379L	IMD, CM	Birth	NA	NA	–	+	–	Pacheco-Cuellar et al., 2017
c.1504G > T/p.D502Y	c.1504G > T/p.D502Y	IMD	6.5 yr	–	1/1	1/1	–	–	Sassi et al., 2014
c.1558G > A/p.A520T	c.1558G > A/p.A520T	IMD	1 yr	NA	NA	NA	NA	NA	Abolhassani et al., 2019
**Compound heterozygous mutations**									
c.715G > C/p.D239H	1.2 Mb incl. entire gene	CDG, IMD, SD	Birth	–	–	–	–	–	Stray-Pedersen et al., 2014
c.1352A > G/p.Q451R	c.737dupA/p.N246K fsX7	CDG, IMD, SD	Birth	1/1	1/1	1/1	1/1	–	Stray-Pedersen et al., 2014
c.1501G > C/p.E501Q	c.1354_1358del CTTAA/p.L452SfsX10	IMD, NI	32 yr	4/5	–	1/5	–	–	Zhang et al., 2014
**Monoallelic mutations**									
c.478C > T/p.P160S	–	BECTS	7 yr	–	–	1/2	–	–	The present study
c.626A > G/p.K209R	–	FAS	1 yr	–	–	–	1/1	–	de la Morena-Barrio et al., 2018
c.1239C > G/p.N413K	–	BECTS	3 yr	–	–	1/1	–	–	The present study
c.1369G > A/p.A457T	–	DD	NA	NA	NA	NA	NA	NA	The Deciphering Developmental Disorders Study, 2017
c.1432C > T/p.Q478X	–	BECTS	12 yr	–	–	1/1	–	–	The present study
c.1659T > A/p.N553K	–	BOE	1 yr	–	–	1/2	–	–	The present study

**The fractions in the table represent the ratio of the number of cases with corresponding symptoms to the total number of cases. IMD, immunodeficiency; CDG, congenital disorder of glycosylation; NI, neurocognitive impairment; CM, congenital malformations; SD, skeletal dysplasia; FAS, fetal alcohol syndrome; DD, developmental disorder; BECTS, benign epilepsy of childhood with centrotemporal spikes; BOE, benign occipital epilepsy; NA, not available; yr, year; mo, month; w, week*.*

In addition, one heterozygous mutation c.626A > G/p.K209R was identified in a patient with fetal alcohol syndrome (de la Morena-Barrio et al., 2018); the other heterozygous mutation c.1369G > A/p.A457T was identified in a patient from a big cohort of developmental disorders by WES (Deciphering Developmental Disorders Study, 2017). No manifestation about immunodeficiency and glycosylation disturbance was mentioned.

## Discussion

*PGM3* gene is located on chromosome 6q14.1–q15 (chr6:83164873-83193936) and encodes phosphoglucomutase 3 (*PGM3*). Previous studies have demonstrated that *PGM3* mutations were associated with severe immunodeficiency and glycosylation disorder, commonly accompanied by neurodevelopmental disorders and occasionally by seizures. In this study, four novel heterozygous *PGM3* variants, including two *de novo* variants, were identified in the cases with IFE and without the manifestation of immunodeficiency or glycosylation disorders. Further analysis indicated that biallelic mutations were associated with severer immunodeficiency or glycosylation disorders, whereas heterozygous mutations were potentially associated with mild phenotypes like IFE. The evidence suggests that *PGM3* gene is potentially a candidate pathogenic gene of epilepsy.

*PGM3*, which catalyzes the conversion of N-acetyl-glucosamine (GlcNAc)-6-phosphate into GlcNAc-1-phosphate, is universally required for the synthesis of UDP-GlcNAc, a sugar nucleotide critical to multiple glycosylation pathways (Stray-Pedersen et al., 2014). The *PGM3* gene is ubiquitously expressed across the whole lifespan (including embryonic period)^1^. In *PGM3*-deficiency mice, homozygous or compound heterozygous hypomorphic alleles caused trilineage cytopenias, even embryonic lethality (Greig et al., 2007), suggesting that *PGM3* is a vital gene for fetal development. In contrast, the heterozygous *PGM3*^+/gt^ and *PGM3*^+/mld1^ mice presented subclinical alterations like reduction of *PGM3* activity in multiple tissues (Greig et al., 2007), indicating a genetic dose effect (quantitative correlation). In humans, *PGM3* mutations were initially associated with IMD-23 characterized by recurrent respiratory, skin infections beginning in early childhood, and notably increased serum IgE in laboratory studies. Recently, *PGM3* mutations have also been identified as a cause of CDG, usually exhibiting severe skeletal dysplasia, congenital malformations, and developmental delay. Among the patients with IMD-23 and CDG, neurodevelopmental abnormalities, including developmental delay, cognitive disability, seizures, and hypotonia, were common. Many patients with *PGM3* gene deficiency need hematopoietic stem cell therapy to save their life. Our analysis demonstrated that the cases with severe immunodeficiency and/or glycosylation disturbance all carried homozygous or compound heterozygous mutations, consistent with the results from biallelic deficiency animal models. Previously, two monoallelic mutations (K209R and A457T) were reported in the patients with fetal alcohol syndrome and developmental disorder, respectively (Deciphering Developmental Disorders Study, 2017; de la Morena-Barrio et al., 2018), and no immunodeficiency was mentioned. In the present study, four monoallelic mutations, including two *de novo* mutations, were identified in patients with pure IFE. One truncating mutation led to haploinsufficiency of *PGM3*. The other three missense mutations were highly conserved across the vertebrates and were predicted to alter hydrogen bond formation with surrounding amino acids. All mutations had no allele frequency in the gnomAD database and were predicted to be damaging by multiple software. Taking together the evidence that *PGM3* gene is expressed in brain and associated with neurodevelopmental disturbance, the *PGM3* gene is suggested to be potentially a candidate pathogenic gene of epilepsy. The genotype–phenotype correlations indicate that biallelic mutations in the *PGM3* gene are associated with severe immunodeficiency and glycosylation disturbance, while heterozygous mutations are potentially associated with mild phenotypes like IFE, in accordance with the findings in the *PGM3* deficiency mice.

*PGM3* protein has four domains arranged in an overall heart shape similar to phosphoglucomutase 1 (PGM1) (Stiers et al., 2017). The catalytic cleft is formed by four loops from each domain. The active site of *PGM3* is located in a large central cleft, formed at the confluence of the four structural domains. The cleft contains (i) phosphoserine 64 that participates in phosphoryl transfer, (ii) the metal-binding loop including the three coordinating aspartates (residues 276, 278, and 280), (iii) a sugar-binding loop, and (iv) the phosphate-binding site (residue 505) (Uniprot database) (Stiers et al., 2016). In the present study, besides the truncating mutation Q478X, all missense mutations were predicted to change the hydrogen bonds with their surrounding amino acids and affect the protein structural stability, potentially impairing enzyme catalytic activity. Mutations P160S and N413K were located within the active center and were associated with typical BECTS. Instead, mutation N553K was identified in a family with BOE with incomplete penetrance. N553K was a distance away from the active center and was less conserved, suggesting less damage effect. Our recent study showed that molecular subregional location was a critical factor to determine the damaging effect that presented a continuous distribution and correlated with the severity of phenotypes (Tang et al., 2019), providing one of the explanations for the less damaging effect and atypical symptoms/incomplete penetrance associated with N553K. However, further studies are required to determine its pathogenicity and association with BOE.

IFE is a group of self-limited epilepsies with infrequent partial seizures and a normal background of EEG. Although both BECTS and BOE are regarded as genetically determined epileptic syndromes, big pedigrees are rare. Majority of patients with BECTS and BOE are sporadic. Incomplete penetrance is potentially one of the explanations. The present study demonstrated that less impaired functions of the *PGM3* gene, i.e., heterozygous mutations, were associated with IFE. Additionally, heterozygous mutations with a less damaging effect were potentially associated with the incomplete penetrance. These findings were consistent with the evidence of quantitative correlation from *PGM3* deficiency models, in which the heterozygous damaging resulted in subclinical abnormalities, such as decrease in enzyme activity and hematological alterations (Greig et al., 2007). In the literature, clinical information about parents in the biallelic cases was not available. Generally, in the patients with BECTS and BOE, epileptiform discharges in EEG will disappear, and epileptic seizures will stop in adulthood. Even in childhood, the epileptic seizures in BECTS and BOE are infrequent and usually occur during sleep, which are usually overlooked. These could be the reasons of incomplete penetrance in the parents with PGM3 heterozygous mutations. It was also found that most early-onset patients with immunodeficiency had no seizure, while the late-onset patients had seizure manifestations, suggesting an age-dependent penetrance. The early-onset patients usually had more severe symptoms and rarely survived to the age of epilepsy occurrence. This may be one of the explanations for a portion of the patients with biallelic mutations having no seizure.

Clinically, the features of BECTS and BOE are often overlapped. Up to 20% patients with BOE have rolandic spikes as those with BECTS (Lada et al., 2003). BECTS and BOE may appear in the same patient at different life periods. In the present study, *PGM3* mutations were identified in both BECTS and BOE, suggesting that BECTS and BOE potentially have similar etiologies. To date, multiple genes have been implicated in IFE. The role of multiple rare heterozygous variants in IFE warrants further study.

In conclusion, we identified four novel heterozygous *PGM3* mutations in patients with IFE without apparent manifestation of immunodeficiency and glycosylation disorder. The genetic and molecular evidence implies that the mutations lead to defect of the *PGM3* gene. Taking together the data from the gene expression profile and the *PGM3* deficiency animal model, it is suggested that *PGM3* gene is potentially a candidate pathogenic gene of epilepsy. Further analysis revealed that previously reported biallelic mutations in the *PGM3* gene were associated with severe immunodeficiency and glycosylation disturbance, while monoallelic mutations were associated with mild phenotypes like IFE, suggesting a quantitative correlation between genetic impairment and phenotypic severity, which help explain the mild symptoms and incomplete penetrance in individuals with IFE.

## Data Availability Statement

The datasets for this article are not publicly available due to concerns regarding participant/patient anonymity. Requests to access the datasets should be directed to the corresponding author.

## Ethics Statement

The Ethics Committee of the Second Affiliated Hospital of Guangzhou Medical University provided ethics approval. Written informed consent to participate in this study was provided by the participants’ legal guardian/next of kin. Written informed consent was obtained from the individuals and minors’ legal guardian for the publication of any potentially identifiable images or data included in this article.

## Author Contributions

X-RL and W-PL contributed to the conception of the study. X-RL contributed to the interpretation of clinical data and drafting of the figures and the manuscript. W-JB, JW, and T-TY examined the patient and participated in drafting of the manuscript. B-ML, D-TL, BT, and W-WD contributed to the collection and analysis of clinical data. Y-WS and TS helped perform the analysis with constructive discussions. W-PL provided critical review and substantially revised the manuscript. All the authors read and approved the manuscript before sending the manuscript to the journal for publication.

## Conflict of Interest

This study received funding from UCB Pharma Ltd. The funder was not involved in the study design, collection, analysis, interpretation of data, the writing of this article or the decision to submit it for publication. The authors declare that the research was conducted in the absence of any commercial or financial relationships that could be construed as a potential conflict of interest.

## Footnotes

1 http://www.gdap.org.cn/genedetail.php?symbol=PGM3
