# Supplementary material for: Heterozygous PGM3 Variants Are Associated With Idiopathic Focal Epilepsy With Incomplete Penetrance
Source: Front Genet. 2020 Oct 15;11:559080. doi: 10.3389/fgene.2020.559080 (PMC7597759; doi:10.3389/fgene.2020.559080)
Supplement: Supplementary file 1 [file Table_1.DOCX]

|  | **Case 1**  **c.478C>T/p.P160S** | **Case 2**  **c.1239C>G/p.N413K** | **Case 3**  **c.1432C>T/p.Q478X** | **Case 4**  **c.1659T>A/p.N553K** |
| --- | --- | --- | --- | --- |
| **SIFT (score)** | Damaging(0.0) | Damaging(0.001) | - | Damaging(0.0) |
| **fitCons(score)** | Damaging(0.732) | Damaging(0.732) | Damaging(0.707) | Damaging(0.706) |
| **Polyphen2 (score)** | Probably-damaging(1.0) | Probably-damaging(1.0) | - | - |
| **LRT (score)** | Deleterious(0.000) | Deleterious(0.000) | Deleterious(0.000) | - |
| **MutationTaster (score)** | Disease-causing(1) | Disease-causing(1) | Disease-causing-automatic(1) | Polymorphism(1) |
| **MutationAssessor(score)** | High(4.35) | High(3.965) | - | - |
| **PROVEAN(score)** | Damaging(-8.0) | Tolerable(-5.81) | - | Tolerable(-0.01) |
| **VEST3(score)** | Damaging(0.975) | Tolerable(0.351) | - | Tolerable(0.139) |
| **MetaSVM(score)** | Damaging(0.776) | Tolerable(-0.290) | - | Tolerable(-1.018) |
| **MetaLR(score)**  **M-CAP(score)** | Damaging(0.760)  Damaging(0.334) | Tolerable(0.336)  Damaging(0.142) | -  - | Tolerable(0.048)  Tolerable(0.003) |
| **CADD(score)** | Damaging(31) | Damaging(27.5) | Damaging(41) | Tolerable(2.766) |
| **DANN(score)** | Damaging(0.999) | Damaging(0.998) | Damaging(0.996) | Tolerable(0.726) |
| **FATHMM_MKL(score)** | Damaging(0.989) | Damaging(0.905) | Damaging(0.993) | Tolerable(0.007) |
| **Eigen(score)** | Damaging(0.954) | Damaging(0.416) | Damaging(1.004) | Tolerable(-0.947) |
| **GenoCanyon(score)** | Damaging(1.000) | Tolerable(0.292) | Damaging(1.000) | Tolerable(0.000) |

**SUPPLEMENTARY TABLE 1 Predictions for the pathogenicity by the in silico programs**
